# Impoverishment Effect of Hydatid Disease and Precision Medical Assistance Pattern of Government: Evidence from Yushu in China

**DOI:** 10.3390/ijerph19169990

**Published:** 2022-08-13

**Authors:** Yaozu Xue

**Affiliations:** School of Humanities and Social Sciences, Xi’an Jiaotong University, Xi’an 710049, China; xueyz2010@xjtu.edu.cn

**Keywords:** Hydatid disease, precision medical assistance pattern, three-level diagnosis and treatment framework, precision poverty alleviation, Yushu Prefecture

## Abstract

Hydatid disease is one of the 17 neglected tropical diseases recognized by WHO and causes a huge global disease burden. Hydatid disease poses a great threat to local medical poverty alleviation. In efforts to break the vicious circle of poverty, Hydatid disease has been widely concerned and discussed. In the practice of poverty alleviation in China, medical poverty alleviation is regarded as the double goal of getting rid of poverty and promoting the construction of a healthy China. On the basis of on-the-spot investigation in Yushu Prefecture, this paper conducts a follow up study on the poverty-causing effect of Hydatid disease and the precision medical assistance pattern of government using a field investigation method. The results show that Hydatid disease led to the increase of poverty in the population in Yushu Prefecture, precision medical assistance played an obvious role in treating Hydatid disease and poverty alleviation, the health service in the study area continues to improve and the medical backbone team further expanded. The main conclusion is that the three-level diagnosis and treatment framework can effectively reduce local poverty and improve people’s living environment.

## 1. Introduction

Hydatid disease is one of the typical endemic diseases in China. As a class of diseases based on regional characteristics, endemic disease has a profound impact on residents in specific areas. According to the World Health Organization, there are about 400 million patients worldwide and more than two billion people are threatened by endemic disease. The occurrence and distribution of various endemic diseases are closely related to local geographical environment, biological species and human production or lifestyle. Endemic disease in China mainly occur in vast rural areas, mountainous areas, pastoral areas, and other remote areas, and the endemic areas are distributed like foci, with a threatened population of more than 420 million. From a regional perspective, endemic disease in China is mostly distributed in the central and western regions. For a long time, endemic disease has been an important cause of poverty in China. The World Bank has two poverty lines: $2 a day for a well-off society and the $1.90 line, called the absolute or extreme poverty line, has been used in more than two dozen of the world’s poorest countries since2015. Bhattacharyya (2016) has explored the causal relationship between geography, diseases, colonies and poverty in developing countries, and found that unfavorable geographical features and endemic disease caused by unfavorable geographical features are important reasons for poverty in Africa. Environmental pollution often leads to disease and poverty [1,2,3,4].

More scholars in China have also found that endemic disease leads to poverty. Li (2020) found that poor people are still prone to endemic disease and their neglect of endemic disease often makes them miss the best treatment opportunity, which not only causes the decline of the labor force of patients, but also leads to the aggravation of poverty [5]. Zheng (2017) evaluated the prevention and control effect of iodine deficiency disorders and drinking water fluorosis in Huzhu County, Qinghai Province. The results show that iodine deficiency disorders and endemic fluorosis in Huzhu County have caused local poverty [6]. Sichuan Provincial Department of Finance and Sichuan Provincial Committee of Agriculture and Industry Joint Investigation Team (2012) investigated the pilot work of poverty alleviation and development and comprehensive prevention and control of Kaschin Beck disease in Aba Prefecture, Sichuan Province. The study found that Aba Prefecture has achieved the pilot objectives of effectively controlling Kaschin–Beck disease and increasing production and income of people in poverty-stricken areas [7]. Wang & Ji (2020) analyzed the distribution of human brucellosis in Qingzhou City from 2011 to 2017. They found that the threat to health posed by human brucellosis led to poverty [8].

China’s precise poverty alleviation strategy always focuses on preventing poverty caused by illness. Wang et al., (2012) introduced that China has established community health clinics and township hospitals to improve the overall basic medical environment under the government-led health care reform plan since 2009 [9]. Leeand Park (2015) studied the Korean government’s medical system and found that the egalitarian medical assistance service developed by the Korean government for local diseases should be accompanied by appropriate contributions [10]. Keith et al., (2013) conducted semi-structured interviews with Kenya’s medical service department, public health, and other medical and health authorities. They found that in order to optimize the medical assistance system for local diseases, Kenya Health Workforce Information System is promoting the certification requirements of Kenyan regulatory agencies, ensuring that health professionals receive the latest professional training and improve the quality of care [11]. Zhang et al., (2022) have studied the relationship between atmospheric environmental policies, air pollution and public health, and found that atmospheric environmental policies help reduce air pollution and thus improve public health [12].

However, there is still a lack of research on the government’s precision medical assistance pattern for endemic disease and the effectiveness of medical assistance. At the same time, the homogenization of medical assistance patterns is common in various places, and there is a lack of multi-level and effective medical assistance pattern for endemic disease [13]. Based on the above problems, the innovation lies in the continuous tracking of the effectiveness of government’s precision medical assistance pattern and the proposal of three-level diagnosis and treatment framework, which has important reference value for other countries/regions to deal with endemic diseases. In summary, the purpose of the current study is twofold, as: (i) we aim to investigate the impact of Hydatid disease on the residents in Yushu Prefecture; and (ii) we aim to elucidate that the government’s precision medical assistance pattern has a significant effect on the treatment of endemic diseases. In this paper, the field investigation method is adopted as the main data collection method, and we used the system analysis method throughout the whole investigation. The results find that Hydatid disease led to the increase of poverty population in Yushu Prefecture, but the precision medical assistance pattern of government played an important role in treating Hydatid diseases and poverty alleviation, especially the three-level diagnosis and treatment framework. In addition, the results also shows that the health service in Yushu Prefecture continues to improve, and the medical backbone team further expanded. Finally, some suggestions were made for the countries/regions in the world plagued by endemic diseases.

## 2. Materials and Methods

### 2.1. Materials

**Figure 1:**
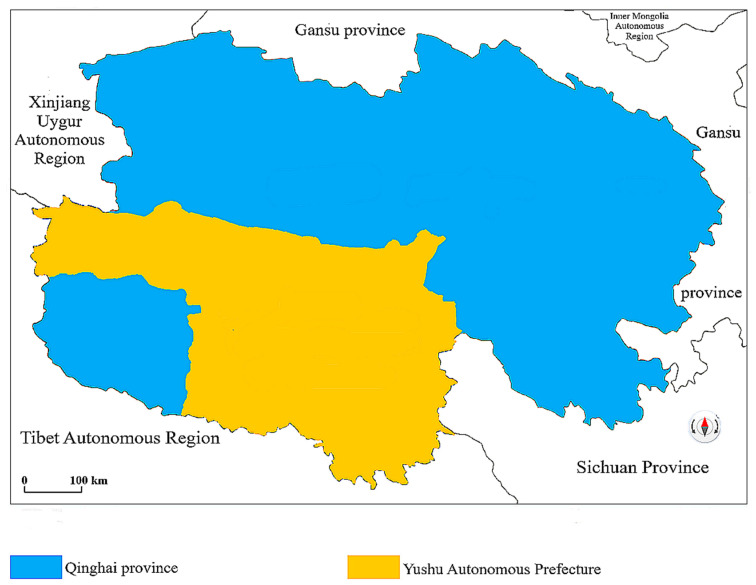
Geographical location map of Yushu Prefecture. Note: Yushu Prefecture generally refers to Yushu Autonomous Prefecture, which is one of the eight prefecture-level administrative regions in Qinghai.

Qinghai Province is located in western China, northeast of Qinghai-Tibet Plateau. Qinghai Province has a total area of 722,300 square kilometers, accounting for one thirteenth of China’s total area. It is the link connecting Tibet, Xinjiang, and the mainland, with an average altitude of over 3000 m. The investigated area is located in Yushu Prefecture, with its capital in Yushu City, is one of the eight prefecture-level administrative districts in Qinghai, and the minority prefecture in Qinghai Province, the total area is 267,000 square kilometers. The geographical location map of Yushu Prefecture is shown as Figure 1.

**Figure 2:**
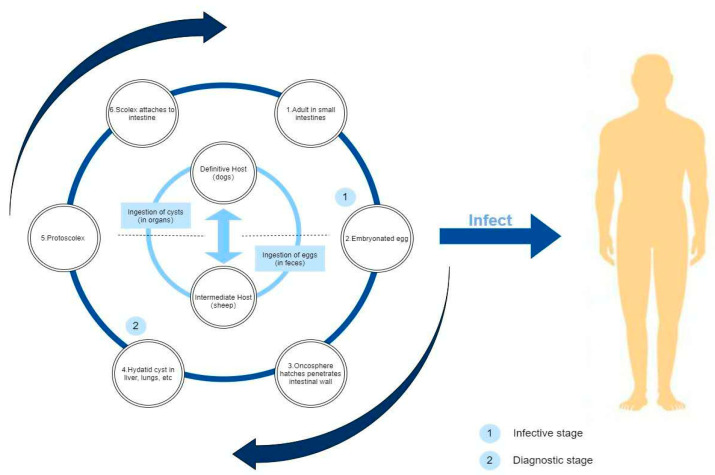
Flow chart of hydatid growth and infection.

**Figure 3:**
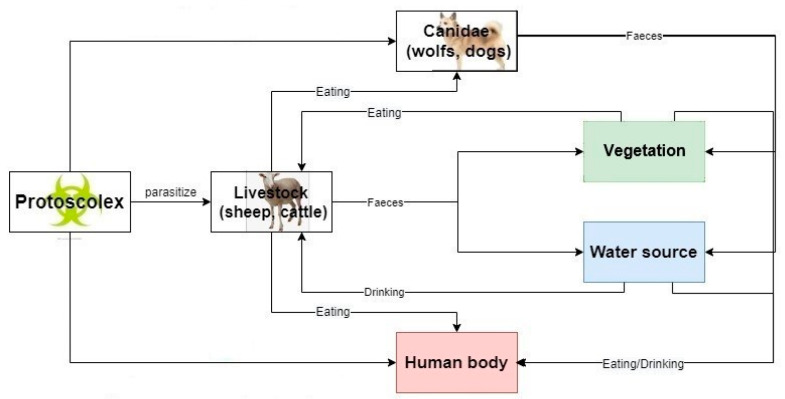
Infection process of hydatid in biological chain.

Due to the topography of Qinghai province, livestock farming is the mainstay of the industry, and most of the residents graze livestock and make a living from it. At the same time, animal breeding is mostly done on a small scale in family workshops, where slaughtered livestock and feed the dogs with raw offal. Thus, the hydatid that causes Hydatid disease is often parasitic on animals such as cattle, sheep and dogs, its eggs are round, with double embryo membranes and tenacious vitality. The eggs of hydatid are excreted with feces, polluting vegetation, soil and drinking water, after being eaten by intermediate hosts such as humans or animals, they enter the duodenum from the stomach. The hydatid eggs come out of the belly of human or animals, drill into the intestinal wall, and then enter the venous system with blood circulation. Most of the larvae are blocked in the liver and develop into adult worm, some hydatid eggs can develop into adult worm in the lungs or through the lungs to organs all over the body [14,15,16]. However, the lifestyle and hygiene habits, as well as the literacy or religious beliefs have played a significant role in the prevalence of the Hydatid disease, causing it to spread rapidly in Qinghai province and making the prevalence of the disease much higher than other province of China. Figure 2 shows the infection process of Hydatid disease and Figure 3 describes the infection process of Hydatid disease in the biological chain.

### 2.2. Research Methods

In this paper, the field investigation method is adopted as the main method. For the literature review, we systematically reviewed the poverty caused by endemic disease and the impact of endemic disease on human health, then obtained the research on the poverty causing effect of endemic disease. Before reviewing the relevant literature, this paper puts forward three research questions: first, what are the causes of poverty and poverty caused by endemic disease; second, what kind of medical assistance pattern is formulated by the Chinese government to reduce the impact of endemic disease on poverty, and how to implement the medical assistance pattern according to local conditions; third, what is the key process of the “win–win” result of poverty reduction? According to the research problems, we summarized that the research should include the poverty-causing effect of endemic disease in Yushu Prefecture and the precise medical assistance pattern of government.

Then, we extract the relevant information from these studies, and formulate the framework according to the characteristics of each medical assistance pattern. According to Fisher et al., (2014), Generic and comprehensive frameworks are valuable thinking tools to apply to a situation, for identifying key elements and processes and demonstrating them in detail. Frameworks are welcomed because they provide checklists analyzing most situations regardless of prior familiarity [17]. Our framework is conducive to a systematic understanding of the precise medical assistance pattern in Yushu Prefecture. It summarizes the experience of this level of medical assistance pattern, Beijing’s counterpart medical assistance pattern and internet online medical assistance pattern, and then systematically analyzes the interrelation between several medical assistance patterns and poverty reduction results.

During the field investigation in Yushu Prefecture, we used the system analysis method throughout the whole investigation, which consists of typical investigation method, comparative analysis method and falsification method. First, in the process of collecting data, the purpose of a typical investigation method is to collect firsthand data from poverty alleviation offices in Qinghai Province, poverty alleviation offices in Yushu Prefecture and demonstration counties of Hydatid disease prevention under Yushu Prefecture. After sorting out and analyzing the data, we established two assumptions:

**H1.**  *Hydatid disease has caused poverty in Yushu Prefecture.*

**H2.**  *The poverty caused by Hydatid disease cannot be alleviated by external assistance.*

Afterwards, we obtained information from conversations with the directors of Related Government Departments, Residents of counties surveyed in Yushu Prefecture and villagers in Chengduo County. By using typical investigation methods, H1 can be proved. Secondly, after collecting the data, this paper uses a comparative analysis method to analyze the detection rates of Hydatid disease and the incidence of poverty, the increasing trend or decreasing trend of poverty due to illness, the cumulative number of screening Hydatid disease, the cumulative number of surgical patients, and the prevalence rate of Hydatid disease in Yushu Prefecture after implementation of the three-level diagnosis and treatment framework etc. Finally, the falsification method refers to disproving the hypothesis by analyzing the effect of medical assistance measures after implementation. This method and comparative analysis method are used to reject H2.

## 3. Results

Based on the field investigation of Yushu Prefecture, this paper made a comparative study on the poverty-causing effect of Hydatid disease and the precise medical assistance pattern of government from the perspective of poverty caused by illness. The results are as follows:

### 3.1. Relationship between the Hydatid Disease and Poverty

**Figure 4:**
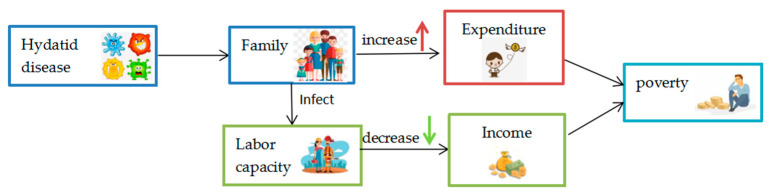
Relationship between the Hydatid disease and poverty.

The typical investigation found that Hydatid disease has caused poverty in two ways: the first is that multiple members of a family infected with Hydatid disease significantly increase the cost of surgical treatment, which may lead the family to poverty; the second is that the health shock caused by the disease reduces people’s ability to work during their illness, and they will not be able to carry out animal husbandry or other labor activities according to seasonal changes [18], which makes the herdsmen who depend on animal husbandry lose their main family economic income and fall into poverty [16].The relationship between the Hydatid disease and poverty is shown in Figure 4. According to the result, H1 can be accepted.

### 3.2. The Detection Rates of Hydatid Disease

The detection rates of Hydatid disease were mainly based on the typical investigation method, and the field investigation was mainly carried out in Chengduo County under the typical areas of Yushu Prefecture in December 2017 and December 2020. The two field investigations covered seven towns in Chengduo County, named Chengwen, Xiewu, Qingshuihe, Zhaduo, Gaduo, Labu and Zhenqin. In 2017, 3450 questionnaires were distributed, and 3427 valid questionnaires were obtained. Excluding invalid ones, the effective rate is 99.33%. In 2020, 3450 questionnaires were distributed, excluding invalid questionnaires, 3438 valid questionnaires were obtained, and thus the effective rate is 99.65%.

Table 1 describes the sample data of detection of Hydatid disease in different towns in December 2017 and December 2020. In 2017, the top three towns of detection rates were Qingshuihe Town, Zhadu Town and Labu Town, with 8.95%, 7.41% and 6.77%, respectively, and the number of detected cases was 51, 34 and 22, respectively. The overall detection rate of Hydatid disease was 6.59%. In 2020, the top three towns of detection rates were Qingshuihe Town, Chengwen Town and Labu Town, with 1.07%, 0.98% and 0.85%, respectively, and the number of detected cases was 6, 6 and 3 respectively. The total detection rate was 0.79%, which was a significant decrease from 2017. The detection rates of Hydatid disease in the respondents in each township in Chengduo County are shown in Table 1. The descriptive statistical results of the main variables are shown in Table 2.

The detection rates in 2017 and 2020 were entered into an Excel, and *t*-test was used to compare the means of the two groups of data. In *t*-test, we put forward following hypothesis: $H_{0}:\mu_{2017}\leq \mu_{2020}$, $H_{1}:\mu_{2017}>\mu_{2020}$.

$$\mathrm{If}\;t_{stat}>t_{critical},\;H_{0}\;should\;be\;rejected.$$

According to Table 3, $t_{stat}$ = 12.81, which is higher than $t_{critical}=2.36$, therefore it can be concluded that $H_{0}\;should\;be\;rejected\;and\;\mu_{2017}>\mu_{2020}$. It can be proved that, with the promotion of precision medical assistance pattern of government, the detection rate of Hydatid disease in Yushu Prefecture decreases from 2017 to 2020.

### 3.3. Effect of Health Services

**Figure 5:**
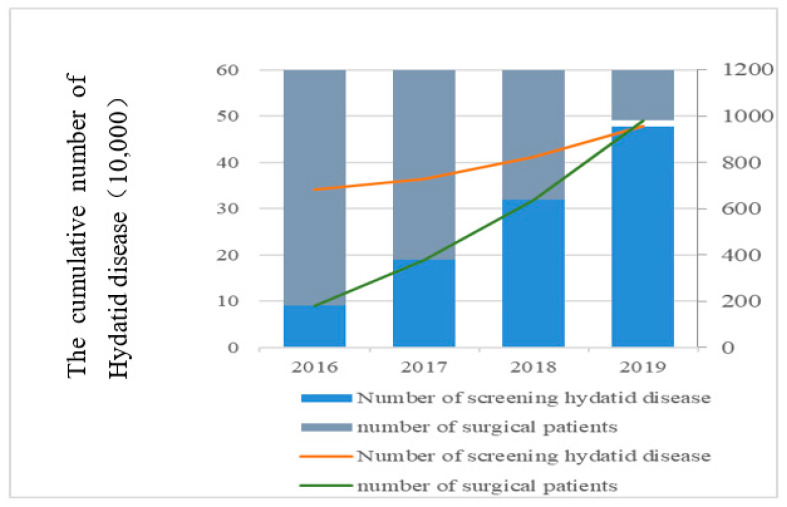
The cumulative number of surgical patients and Hydatid disease screened in Yushu Prefecture from 2016 to 2019.

According to the interview from poverty alleviation offices in Qinghai Province, poverty alleviation offices in Yushu Prefecture and demonstration counties of Hydatid disease prevention under Yushu Prefecture, we collect the implementation of the 13th Five-Year Plan for Prevention of Major Infectious Diseases and Endemic disease in Yushu Prefecture (2016–2020) and the Action Plan for Prevention and Control of Hydatid disease in Yushu Prefecture (2016–2020). Since 2016, it has integrated funds of ¥140 million by Yushu Prefecture to implement the first health census of residents with Hydatid disease screened in Tibetan areas [19]. The cumulative number of Hydatid disease screened and the cumulative number of surgical patients are shown in Figure 5.

The government of Yushu Prefecture has actively implemented the free treatment project for Hydatid disease and distributed 692,000 tablets of albendazole and 565,000 milliliters of albendazole emulsion. At the same time, in order to minimize the burden on patients, the government of Yushu Prefecture has also coordinated with the Red Cross Society and the Civil Affairs Bureau on implementing the medical insurance assistance policy for surgical patients of Hydatid disease.

### 3.4. Scale of Medical Backbone Team

**Figure 6:**
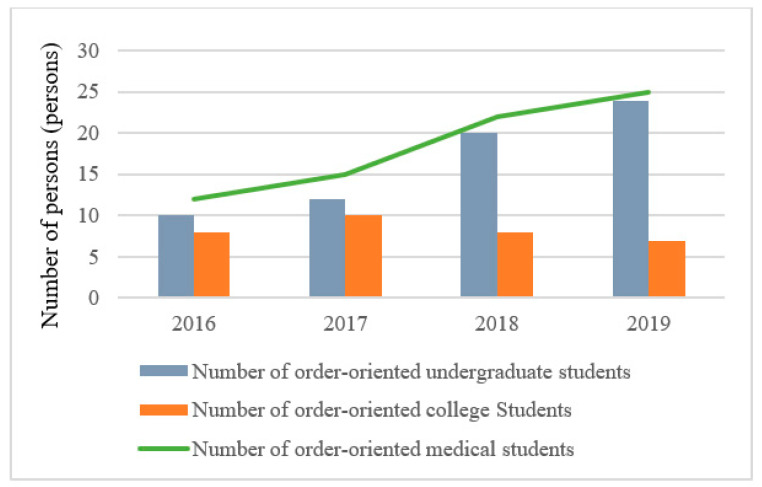
Number of order-oriented medical students recruited in Yushu Prefecture from 2016 to 2019.

The data from the typical investigation shows that the government of Yushu Prefecture takes the form of order-oriented training to continuously strengthen the construction of human resources in various medical institutions. In recent years, more than 180 professionals have been recruited, including 66 undergraduate students, and 22 college students with order orientation. The increase in the number of order-oriented medical students can be seen in Figure 6. At the same time, 11 medical backbones have sent to universities such as Capital Medical University for professional knowledge training, and 120 technical backbones have arranged to go to various support hospitals for the training, which significantly improved the service ability of surgical treatment in each supported hospital of Yushu Prefecture.

### 3.5. Precision Medical Assistance Pattern of Government

**Figure 7:**
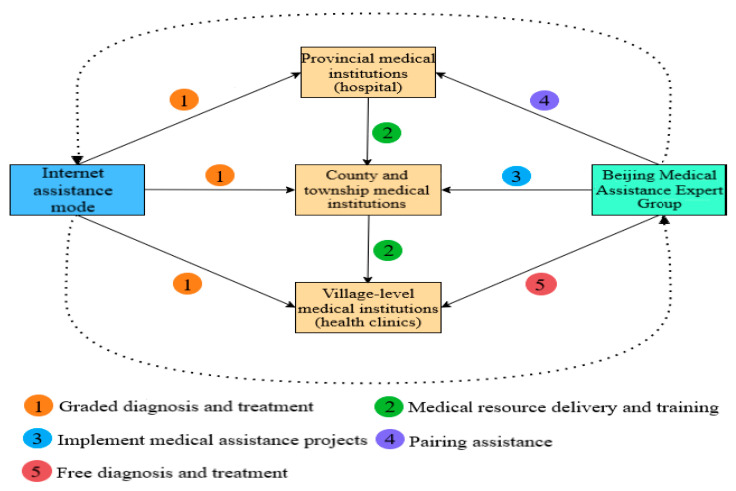
The detailed instructions of three-level diagnosis and treatment framework.

After long-term exploration and practice of China’s targeted poverty alleviation policy, precision medical assistance pattern of Yushu Prefecture was formed, this can be generalized as a three-level diagnosis and treatment framework. It refers to an all-round diagnosis and treatment system with medical assistance pattern as the main pattern and Beijing-Qinghai internet medical assistance pattern as the auxiliary pattern. The three- level diagnosis and treatment framework are divided into three specific patterns: the first is the mutual medical assistance pattern at the same level of provincial-county and township-village medical institutions; The second is the longitudinally medical assistance pattern of “Dean + Team” of medical and health institutions in Qinghai with Beijing medical team; The third is the horizontal cooperative medical assistance pattern of “1 village cadre + 1 medical staff + N patients”. The three-level diagnosis and treatment framework is shown in Figure 7.

## 4. Discussion

### 4.1. Effectiveness of Medical Assistance in Poverty Alleviation

**Figure 8:**
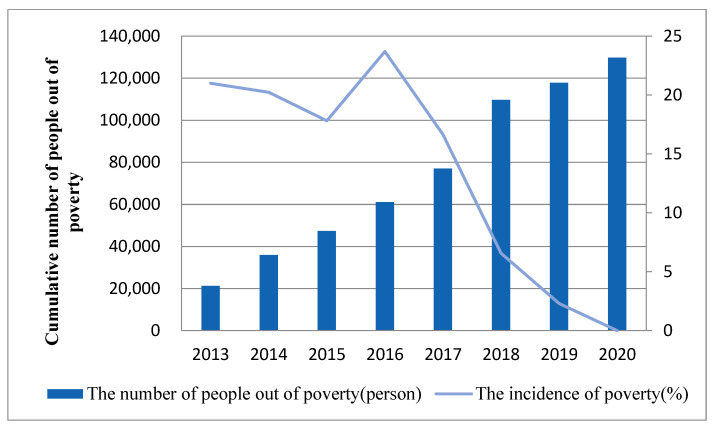
The incidence of poverty and cumulative number of people out of poverty in Yushu Prefecture from 2013 to 2020.

With the promotion of the Precision Medical Assistance Pattern, the effect of Yushu Prefecture’s medical poverty alleviation was significant and had achieved absolute poverty alleviation in 2020. The number of people poverty-returning due to illness in Yushu Prefecture decreased from 78,000 in 2016 to 12,000 in 2019. After the implementation of the three-level diagnosis and treatment framework, the incidence of absolute poverty in Yushu Prefecture has dropped from 20.03% in 2014 to 2.1% in 2019, especially since 2017, the incidence of absolute poverty in Yushu Prefecture has shown a steady downward trend. By the end of 2020, the incidence of absolute poverty in Yushu Prefecture was reduced to zero. Figure 8 shows the trends of incidence of absolute poverty and the cumulative number of people out of absolute poverty in Yushu Prefecture. The changing trends can also prove that Hydatid disease is related to poverty in Yushu Prefecture.

### 4.2. A Three-Level Diagnosis and Treatment Framework

#### 4.2.1. Longitudinally Medical Assistance with Dean as the Core, Team as the Focus and Reform as the Goal

**Figure 9:**
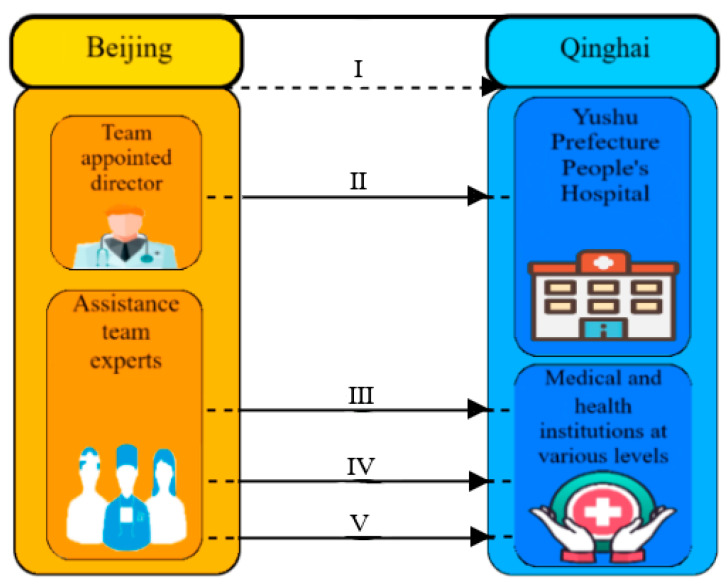
“Dean + Team” medical assistance pattern. Note: I Irregular rotation of medical teams; II Long-term treatment of critically ill patients; III Add new disciplines and opening new wards; IV Train medical assistance team; V Implement medical assistance projects.

“Dean+Team” is an important part of Yushu Prefecture’s three level diagnosis and treatment framework. As the longitudinally medical assistance pattern, Beijing counterpart support expert group guides Yushu Prefecture People’s Hospital to set up a new discipline, promotes the improvement of the overall medical capacity, and provides more convenient medical assistance for patients [20,21]. At present, Yushu Prefecture People’s Hospital has added several new disciplines and opened various new wards. At the same time, it has formed a local disease prevention and control system in counterpart support areas to promote the prevention, treatment and tracking of major diseases by local governments; In addition, Beijing counterpart support medical team carried out assessment and prevention research of altitude sickness, laying a good foundation for the long-term medical construction of Yushu Prefecture. Otherwise, the medical team consolidated the medical assistance pattern of “Dean+Team” and gradually expanded the aid field to disease control, maternal and child health, mental health and many other fields, which not only improved the medical technology, but also improved the public health service capacity of Yushu Prefecture. Figure 9 shows the detailed instructions of “Dean+Team” medical assistance pattern.

#### 4.2.2. Horizontal Medical Assistance of “1 Village Cadre + 1 Medical Staff + N Patients”

**Figure 10:**
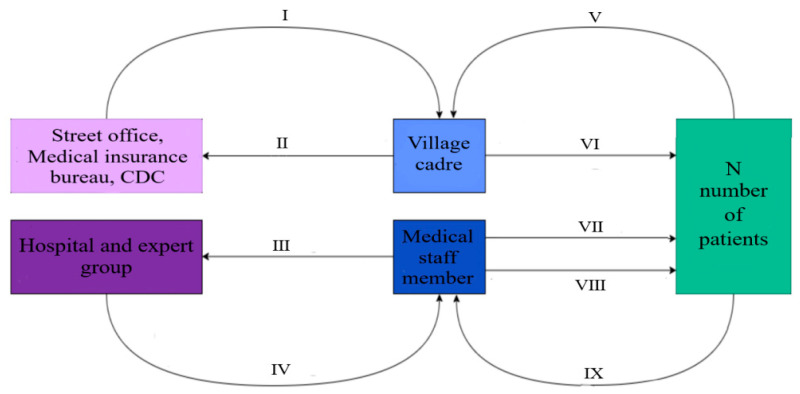
“1 village cadre + 1 medical staff + N patients” assistance pattern. Note: I Assign a filing list; II Summarize the latest health records of residents; III Summarize the latest condition of patients; IV Conduct a diagnosis and treatment plan; V Report health status in time; VI Establish and improve residents’ health records; VII Compare disease data; VIII Provide the treatment and psychological counseling; IX Feedback the rehabilitation situation.

The horizontal medical assistance of “1 village cadre + 1 medical staff + N patients” in Yushu Prefecture is mainly that village cadres set up dcresidents’ health records for patients and appointed medical staff to carry out diagnosis and treatment or psychological counseling for patients, which provides multi-angle help for timely treatment and later rehabilitation. The horizontal medical assistance of “1 Village Cadre + 1 Medical Staff + N Patients” can promote medical assistance in a timely, effective and long-term manner [22]. As shown in Figure 10.

### 4.3. Internet Online Medical Assistance

**Figure 11:**
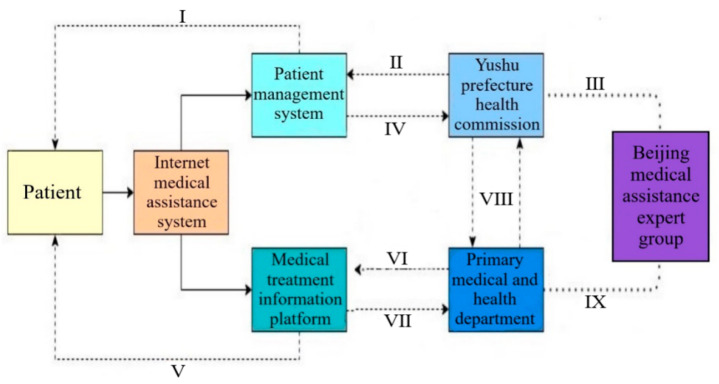
The Flowchart of Beijing-Qinghai Internet Online Medical Assistance. Note: I Implement precise management; II Logging data; III Consultation and treatment; IV Visualized patient condition analysis; V Implement efficient diagnosis and treatment; VI Master patient’s situation; VII Organize, guide and advance; VIII Data sharing; IX Consultation and treatment.

Internet online medical assistance promotes the spread of advanced medical technology and experience in order to improve the medical level in the aided areas. Compared the government-led health care reform plan introduced by Wang, Y. et al., (2012) that China has established community health clinics and township hospitals to improve the overall basic medical environment [8], Internet medical assistance system introduces intelligent Hydatid disease patient management system and implements accurate management for all Hydatid disease patients. It should further strengthen the archives arrangement of Hydatid disease patients after inputting the baseline data and comparative data of Hydatid disease patients in the intelligent Hydatid disease patient management system. Through the visual analysis of the data by the system, it is beneficial to further study the patient’s condition and conduct practical diagnosis and treatment plans for patients, then share the diagnosis and treatment plans with the health departments of city, county, township, and village, so as to improve the diagnosis and treatment efficiency [23]. Especially when encountering intractable diseases, the primary medical and health department can consult and treat them remotely with Beijing counterpart support expert group. The characteristics of hierarchical construction, sub-object management and collaborative diagnosis and treatment of the Internet medical assistance system form a closed-loop Beijing-Qinghai Internet online medical assistance, and the specific flow chart is shown in Figure 11.

## 5. Conclusions

In this paper, Through the field investigation, we find that Hydatid disease has caused the increase of poverty population in Yushu Prefecture, but the precision medical assistance pattern played an important role in treating Hydatid disease and poverty alleviation, which can be generalized as three-level diagnosis and treatment framework. The framework consists of three parts: the first is the mutual medical assistance at the same level with the provincial-county and township-village medical institutions; The second is the longitudinally medical assistance pattern, the medical and health institutions at all levels in Qinghai has been linked with Beijing medical team, and Beijing counterpart support expert group guides Yushu Prefecture People’s Hospital to promote the prevention, treatment and tracking of major diseases; The third is the horizontal medical assistance pattern of “1 village cadre + 1 medical staff + N patients”, which promote medical assistance in a timely, effective and long-term manner in Yushu Prefecture. The three-level diagnosis and treatment framework established in Yushu Prefecture has benefited local patients and effectively achieved the government’s goal of precision medical assistance.

## 6. Suggestions

Our findings suggest that three-level diagnosis and treatment framework in Yushu Prefecture has successfully solved the problem of poverty caused by endemic diseases. The success experience provided lessons for other countries and regions in the world, such as three-level diagnosis and treatment framework, it can be extended to other countries or regions suffering from endemic diseases. For example, it can be applied to the treatment of brucellosis, fluorosis, arsenic, and other endemic diseases.

Further research should be devoted to the improvement of the three-level diagnosis and treatment framework to benefit more countries or regions in the world, and the improvement mainly lies in science and technology, such as digital technology and AI. With the deepening of the integration of internet technology into the field of precision medical assistance pattern of government, the internet medical assistance system will provide patients with more comprehensive and effective assistance. The health departments of city, county (district), township and community (village) use the telemedicine information platform to grasp the patient’s situation and share data in time, the real-time and intuitive “hand-in-hand” clinical guidance of the system greatly improves the diagnosis and treatment efficiency and improves the treatment effects.

## Figures and Tables

**Table 1:** The detection rates of Hydatid disease of the respondents in each town.

Town Name	Respondents Nummer		Case		Detection Rate (%)	
Year	2017	2020	2017	2020	2017	2020
Chengwen Town	623	615	41	6	6.58	0.98
Xiewu Town	456	458	23	3	5.04	0.66
Zhenqin Town	562	571	32	4	5.69	0.70
Qingshuihe Town	570	562	51	6	8.95	1.07
Zhaduo Town	459	452	34	2	7.41	0.44
Gaduo Town	432	429	23	3	5.32	0.70
Labu Town	325	351	22	3	6.77	0.85
Sum	3427	3438	226	27	6.59	0.79

**Table 2:** Descriptive statistics for key variables.

Variable	Mean	Median	Minimum	Maximum
Respondents number in 2017	489.57	456	325	623
Respondents number in 2020	491.14	458	351	615
Case in 2017	32.29	32	22	51
Case in 2020	3.86	3	2	6
Detection rate in 2017 (%)	6.54	6.59	5.04	8.95
Detection rate in 2020 (%)	0.77	0.70	0.44	1.07

**Table 3:** *t*-Test: Two-Sample Assuming Unequal Variances.

	2017	2020
Mean	6.54375	0.77375
Variance	1.58325536	0.03879821
Observations	8	8
Hypothesized Mean Difference	0	
df	7	
t Stat	12.814103	
P(T ≤ t) one-tail	2.0441 × 10^−6^	
t Critical one-tail	1.89457861	
P(T ≤ t) two-tail	4.0882 × 10^−6^	
t Critical two-tail	2.36462425	

## Data Availability

The data are restricted to the Relevant Government Departments in Qinghai Province and cannot be provided to others.
